# Serologic Survey of Hantavirus Infection, Brazilian Amazon

**DOI:** 10.3201/eid1605.090766

**Published:** 2010-05

**Authors:** Wellington S. Mendes, Antônio A.M. da Silva, Romerito F. Neiva, Nicolle M. Costa, Maressa S. de Assis, Priscila M.O. Vidigal, Maria da G. L. Leite, Elizabeth S.T. da Rosa, Daniele B. de A. Medeiros, Darlene de B. Simith, Pedro F. da C. Vasconcelos

**Affiliations:** Federal University of Maranhão, São Luís, Brazil (W.S. Mendes, A.A.M. da Silva, R.M. Neiva, N.M. Costa, M.S. de Assis, P.M.O. Vidigal); Maranhão State Department of Health, São Luís (M.G.L. Leite); Evandro Chagas Institute, Belém, Brazil (E.S.T. Rosa, D.B. Medeiros, D.B. Simith, P.F.C. Vasconcelos); 1 Deceased.

**Keywords:** Viruses, hantavirus, Brazil, serology, surveillance, Amazon, letter

**To the Editor**: Since the etiology of hantavirus cardiopulmonary syndrome (HCPS) was recognized in 1993 in the United States (*1*), various hantaviruses have been associated with the syndrome in South America (*2*,*3*). Depending on the viral genotype involved, hantavirus infection can take a wide variety of forms, from asymptomatic or oligosymptomatic to the classic clinical form (*4*,*5*).

The first cases of HCPS in Brazil were reported in the state of São Paulo in 1993 (*6*,*7*). In 2000, an outbreak of HCPS was reported in the municipality of Anajatuba in the state of Maranhão in the Maranhão western lowlands, a microregion in the Brazilian Amazon (*8*,*9*). An ecologic study in this region identified antibodies against hantaviruses in wild rodents (*Oligoryzomys fornesi* and *Holochilus sciureus*). Analysis of RNA from the viruses isolated from these 2 rodent species showed 2 new hantaviruses, which were named Anajatuba and Rio Mearim, respectively (*10*).

The Maranhão western lowlands is a swampy region that consists of 21 municipalities and ≈400,000 inhabitants. A cross-sectional study was conducted from August 2004 through September 2006 to identify exposures and activities associated with hantavirus infection. A convenience sample comprising 6 of the 21 municipalities in the region was selected: Pinheiro in the north, Vitória do Mearim in the south, São Bento and São João Batista in the east, and Penalva and Viana in the west. In each municipality, a village in the countryside was randomly chosen. All members of those communities were invited to participate. Persons who agreed to take part in the study were interviewed, and a standardized questionnaire was used to collect demographic information. Participants provided blood samples after giving written informed consent. ELISA to detect immunoglobulin (Ig) G antibodies against hantaviruses was performed in the Arbovirology and Hemorrhagic Fevers Department at the Evandro Chagas Institute (Belém, PA, Brazil) by using antigen from the Sin Nombre virus supplied by the Centers for Disease Control and Prevention (Atlanta, GA, USA). Samples were initially screened at 1:100, and all positive results were confirmed in a serial dilution (starting at 1:400) test. Positive samples were those with titers >400 (*10*).

Odds ratios (ORs) were estimated by logistic regression. Variables with p<0.20 in the univariate analysis were subjected to multivariate analysis.

Our study comprised 1,386 persons. The seroprevalence of antihantavirus antibodies in the 6 municipalities was 4.7% (65/1,389), distributed as follows: São João Batista, 2.6% (8/307); São Bento, 3.1% (6/195); Penalva, 3.5% (5/145); Pinheiro, 4.8% (13/273); Viana, 5.6% (9/160); and Vitória do Mearim, 7.8% (24/309). Most persons interviewed were farmers and illiterate and lived in rammed-earth huts. Killing rats in the fields was reported by 24.3% (337/1,389). According to univariate analysis, seeing rats in the dwelling or in the field, having killed rats in the dwelling or in the fields, being a farmer, being male, and being >20 years of age were associated with IgG against hantaviruses. No association was found between storage of grain inside a dwelling or presence of natural predators of rodents, e.g., domestic cats and snakes such as the red-tailed boa, and antibodies against hantaviruses (Table).

**Table:** Univariate analysis of risk factors associated with hantavirus infection in humans, Maranhão western lowlands, Brazil, 2006*

Possible risk factor	SNV IgG positive, no. (%), n = 65	SNV IgG negative, no. (%), n = 1,324	OR (95% CI)	p value
Storing grain in the house	38 (58.5)	774 (58.5)	1.00 (0.60–1.66)	0.999
Sweeping the house	53 (81.5)	1176 (88.8)	0.56 (0.29–1.06)	0.076
Seeing rats in the dwelling	47 (72.3)	742 (56.0)	2.05 (1.18–3.56)	0.011
Seeing rats in the fields	41 (63.1)	618 (46.7)	1.95 (1.17–3.27)	0.011
Having killed rats in the dwelling	46 (70.8)	686 (51.8)	2.25 (1.31–3.88)	0.004
Having killed rats in the fields	29 (44.6)	308 (23.3)	2.66 (1.60–4.40)	<0.001
Being bitten by a rat	5 (7.7)	46 (3.5)	2.32 (0.89–6.04)	0.086
Seeing rat feces in the dwelling	34 (52.3)	609 (46.0)	1.29 (0.78–2.12)	0.320
Being a farmer	40 (61.5)	467 (35.3)	2.94 (1.76–4.90)	<0.001
Using rat meat as fishing bait	1 (1.5)	13 (1.0)	1.58 (0.20–12.2)	0.664
Seeing buffaloes in the fields	26 (40.0)	518 (39.1)	1.04 (0.62–1.72)	0.888
Keeping a snake in the house	0	27 (2.0)	Not calculated	0.245
Keeping a cat in the house	33 (50.8)	689 (52.0)	0.95 (0.58–1.56)	0.841
Keeping a dog in the house	39 (60.0)	890 (67.2)	0.73 (0.44–1.22)	0.229
Living in a dwelling close to the fields	47 (72.3)	868 (65.6)	1.37 (0.79–2.39)	0.264
Living near fruit trees	40 (61.5)	829 (62.6)	0.96 (0.57–1.59)	0.861
Living near the forest	29 (44.6)	599 (45.2)	0.98 (0.59–1.61)	0.921
Living in a house >30 m from the fields	23 (35.4)	477 (36.0)	0.97 (0.68–1.64)	0.916
Bathing in flooded fields	27 (41.5)	429 (32.4)	1.48 (0.89–2.46)	0.128
Fishing in flooded fields	43 (66.2)	797 (60.2)	1.29 (0.76–2.19)	0.339
Being age 20–29 y	12 (18.5)	266 (20.1)	7.31 (2.04–26.1)	0.002
Being age 30–39 y	8 (12.3)	226 (17.1)	5.73 (1.51–21.8)	0.010
Being age >40 y	42 (64.6)	346 (26.1)	19.7 (6.05–64.0)	<0.001
Being male	38 (58.5)	590 (44.6)	1.75 (1.06–2.90)	0.030

*SNV, Sin Nombre virus; Ig, immunoglobulin; OR, odds ratio; CI, confidence interval.

Because of colinearity between rat exposure variables, only “having killed rats in the field” was submitted to adjusted analysis. In the multivariate analysis, being >20 years of age and having killed rats in the field remained associated with hantavirus infection. In the model that did not consider sex and age, being a farmer (OR 2.63, 95% confidence interval [CI] 1.56%–4.41%) and having killed rats in the field (OR 2.30, 95% CI 1.38%–3.84%) were independently associated with hantavirus infection.

Hantavirus infection is endemic to the Maranhão western lowlands. It has characteristics of an occupational infection associated with agricultural work, but age mostly explained the effect of being a farmer (OR for being a farmer 1.29, 95% CI 0.74%–2.24%, adjusted for sex and age). Exposure to rodents appeared to occur both in fields and dwellings.

Although serologic evidence indicated that hantaviruses were circulating in the 6 municipalities studied, no cases of HCPS were reported in any of them. This finding suggests that either only mild or asymptomatic cases were occurring and that these were not recognized as HCPS or that cases of hantavirus were not being detected and reported. However, in a study in 2000 in Anajatuba in a cohort of 234 persons who did not initially have IgG against hantaviruses, 4 seroconversions to IgG against these viruses were detected; 2 of these infections were asymptomatic and 2 were self-limiting febrile illnesses that occurred after 24 months of follow-up (*9*). The present study reinforces the suspicion that mild or atypical cases are occurring in this region and may be the main reason classical hantavirus infection has not been identified.
